# An integrative bioinformatics investigation and experimental validation of chromobox family in diffuse large B-cell lymphoma

**DOI:** 10.1186/s12885-023-11108-6

**Published:** 2023-07-10

**Authors:** Fenling Zhou, Lu Chen, Peng Lu, Yuli Cao, Cuilan Deng, Gexiu Liu

**Affiliations:** 1 Institute of Hematology, Jinan University, HuangPu Da Dao Xi, Guangzhou, Guangdong 510632 People’s Republic of China; 2 Departpent of Vascular Surgery, The Second Xiangya Hospital, Central South University, Hunan Province, No. 139, Renmin Road, Changsha, China; 3 Department of Hematology, First Affiliated Hospital, Jinan University, HuangPu Da Dao Xi, Guangzhou, Guangdong 510632 People’s Republic of China

**Keywords:** DLBCL, Chromobox family, Bioinformatics analysis, Biomarker

## Abstract

**Background:**

Diffuse large B-cell lymphoma (DLBCL) is one of the most aggressive malignant tumors. Chromobox (CBX) family plays the role of oncogenes in various malignancies.

**Methods:**

The transcriptional and protein levels of CBX family were confirmed by GEPIA, Oncomine, CCLE, and HPA database. Screening of co-expressed genes and gene function enrichment analysis were performed by GeneMANIA and DAVID 6.8. The prognostic value, immune cell infiltration and drug sensitivity analysis of CBX family in DLBCL were performed by Genomicscape, TIMER2.0, and GSCALite database. Confirmatory Tests of CBX family protein expression in DLBCL were performed by immunohistochemistry.

**Results:**

The mRNA and protein expressions of CBX1/2/3/5/6 were higher in DLBCL tissues than control groups. Enrichment analysis showed that the functions of CBX family were mainly related to chromatin remodeling, methylation-dependent protein binding, and VEGF signaling pathway. The high mRNA expressions of CBX2/3/5/6 were identified to be associated with short overall survival (OS) in DLBCL patients. Multivariate COX regression indicated that CBX3 was independent prognostic marker. Immune infiltration analysis revealed that the mRNA expressions of CBX family (especially CBX1, CBX5, and CBX6) in DLBCL were significantly correlated with the infiltration of most immune cells (including B cells, CD8 + T cells, CD4 + T cells, neutrophils, monocytes, macrophages, and Treg cells). Meanwhile, there was a strong correlation between the expression levels of CBX1/5/6 and surface markers of immune cells, such as the widely studied PVR-like protein receptor/ligand and PDL-1 immune checkpoint. Notably, our study found that DLBCL cells with CBX1 over-expression were resistant to the common anti-tumor drugs, but CBX2/5 had two polarities. Finally, we confirmed the higher expressions of CBX1/2/3/5/6 in DLBCL tissues compared with control groups by immunohistochemistry.

**Conclusion:**

We provided a detailed analysis of the relationship between the CBX family and the prognosis of DLBCL. Distinguished from other studies, We found that high mRNA expressions of CBX2/3/5/6 were associated with poor prognosis in DLBCL patients, and Multivariate COX regression indicated that CBX3 was independent prognostic marker. Besides, our study also found an association between the CBX family and anti-tumour drug resistance, and provided a relationship between CBX family expression and immune cell infiltration.

**Supplementary Information:**

The online version contains supplementary material available at 10.1186/s12885-023-11108-6.

## Introduction

Diffuse large B-cell lymphoma (DLBCL) is the main subtype of non-Hodgkin’s lymphoma, with clinical and genetic heterogeneity characteristics [1, 2]. Existing clinical chemotherapy regimens could achieve survival rates of 50–60% in DLBCL patients. However, due to the heterogeneity of the malignancy, about 40% of patients did not fully benefit [3]. Personalized therapy of targeted oncogenes may be more precise than chemical immunotherapy, but it is prone to drug resistance [4]. Most treatment strategies target tumor cells directly; however, the genetic stability of stromal cells and immune cells in the tumor microenvironment (TME) can avoid the impact of treatment resistance [5].

Chromobox (CBX) family is the canonical component of the polycomb inhibitory complex, an epigenetic regulatory complex that modifies chromatin to transcriptionally inhibit target genes [6]. It has been reported that the abnormal expression of CBX family had important prognostic value in various tumors. In liver cancer, CBX1/2/3/6/8 were found to be a prognostic biomarker [7]. In breast cancer, Zeng et al. [8] reported that CBX4 exerted oncogenic activity through the Notch1 signaling pathway. The high expressions of CBX3, CBX4, and CBX5 in lung cancer were also related to the poor prognosis of patients [9–11]. However, the unique role of CBX family in DLBCL is unclear.

Bioinformatics is often used to find key genes associated with specific biological processes. Through the utilization of serial bioinformatics analysis, Liu et al. [12] developed a prognostic prediction model based on long non-coding RNAs associated with tumor stemness. Their findings indicated that stemness-associated genes could regulate the apoptotic signaling pathway, thereby influencing tumor progression. Furthermore, the prognostic signature of stemness-associated genes showed promise as diagnostic and prognostic biomarkers for renal clear cell carcinoma. Jiang et al. [13] conducted bioinformatic analyses on abnormally expressed RNA-binding proteins (RBPs) implicated in tumorigenesis, invasion, and prognosis. Whereafter, they identified six RBPs that were strongly related to the prognosis of renal papillary cell carcinoma. These findings led to the establishment of a six-RBP prognostic model, which improved the predictive ability of the staging system and enabled the prediction of overall survival (OS). In their comprehensive analysis combining meta-analysis and bioinformatics, Zhang et al. [14] provided evidence supporting the association between mutations in DNA damage response pathways and an unfavorable prognosis in patients with prostate cancer. And they identified specific gene mutations, including ATR, BLM, and MLH1, that could potentially enhance the sensitivity to Olaparib. Through a series of meticulous bioinformatics analyses and rigorous verification experiments, Yu et al. [15] unveiled the overexpression of complement and its positive correlation with tissue factor in Endometriosis. These findings suggested that the interplay between complement and coagulation may have a pivotal role in the development of Endometriosis.

**Fig. 1:**
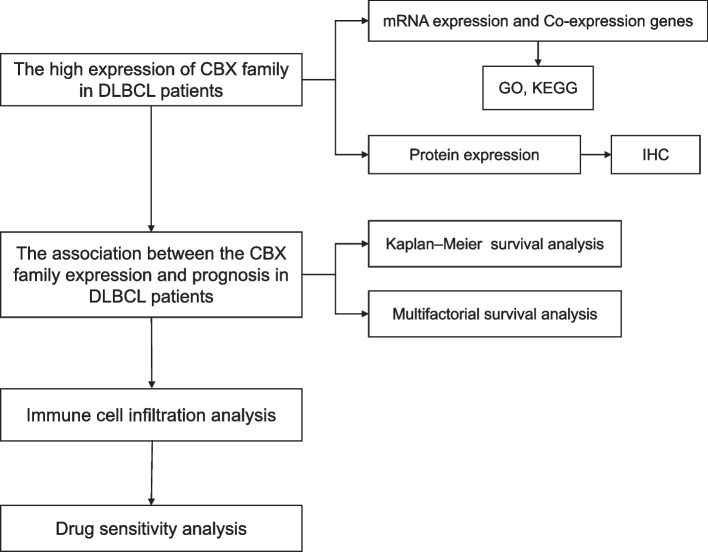
The flow diagram of the whole study

Although a preliminary bioinformatics study on the relationship between the CBX family and DLBCL has been published recently, there were still some shortcomings, such as too few clinical survival samples, in addition, some clinically relevant questions remain unanswered, such as the prognosis analysis based on multifactorial analysis. Our study provided a detailed analysis of the relationship between CBX family expression and the prognosis of DLBCL patients. We also conducted a comprehensive analysis of the mRNA/protein expression, immune infiltration, and drug sensitivity of CBX family in DLBCL. Meanwhile, immunohistochemistry was performed to verify the protein expression of CBX family in DLBCL tissues. The flow chart of this study was shown in Fig. 1.

## Methods

### The mRNA expression analysis of CBX family in DLBCL

Gene Expression Profiling Interactive Analysis (GEPIA, www.gepia.cancer-pku.cn), a newly developed interactive web server that contains the RNA expression of 9,736 tumors and 8,587 normal samples from the Cancer Genome Atlas and the Genotype Tissue Expression projects [16]. The dataset could provide customizable functions including differential expression analysis, correlation analysis, patient survival analysis, etc. The mRNA expressions of CBX family were analyzed by GEPIA. We utilized ‘Expression on Box Plots’ module to detect the mRNA expressions of CBX family members in DLBCL patients from GEPIA database. *P*-value < 0.05 was considered statistically different.

Oncomine (http://www.oncomine.org) is an online cancer microarray database containing 715 data sets and 86,733 samples, designed to collect, standardize, analyze, and provide cancer transcriptome data to the biomedical research community [17]. A Students’ t-test was performed to conduct a comparative analysis of the transcriptional expressions of CBX family between DLBCL tissues and normal controls. Analysis type: cancer vs. normal analysis; data type: mRNA. The threshold *P*-value was defined as 0.05.

The CCLE dataset (https://www.Broadinstitute.org/ccle) is a compilation of gene expression, chromosomal copy numbers, and massively parallel sequencing data from about 1,000 cell lines [18]. The mRNA expressions of CBX family in cancer cell lines (including DLBCL) were assessed by the CCLE dataset. Box-and-whisker plots showed the distribution of CBX family expression for each tumor subtype, ordered by the median CBX family expression level (line), the inter-quartile range (box) and up to 1.5 × the inter-quartile range (bars). Sample numbers were indicated in parentheses. Subsequently, mRNA expressions of CBX family members in DLBCL cell lines were verified by the EMBL-EBI dataset (https://www.ebi.ac.uk) [19]. Darker blue means higher gene expression.

### Screening of co-expressed genes and gene function enrichment analysis

GeneMANIA (http://www.genemania.org) is a flexible, user-friendly web interface, which generates hypotheses about gene function prediction and detects genes with similar functions [20]. In this study, we constructed a gene–gene interaction network for CBX family using the GeneMANIA database (http://genemania.org/). The species Homo sapiens was selected, and the genes chosen were the CBX family members (including CBX1/2/3/5/6/8). We obtained a comprehensive collection of genes that had close associations with the CBX family in terms of sharing protein domains, prediction, physical interactions, and co-expression. Subsequently, we selected the top 100 genes with the highest relevance for further analysis. Then, the Gene Ontology (GO) and the Kyoto Encyclopedia Gene and Genome (KEGG) pathway enrichment analysis of CBX family and their co-expressed genes were performed using DAVID 6.8 (https://david.ncifcrf.gov/home.jsp) [21]. GO enrichment analysis predicted the functions of genes in three aspects, including biological process (BP), cellular component (CC), and molecular function (MF). The critical value was a false discovery rate (FDR) of < 0.05.

### The protein expression analysis of CBX family in DLBCL

The Human Protein Atlas (HPA) is a free public repository that contains the protein expression data of 17 different human cancers (detected by immunohistochemistry), which was used to study the protein expressions of CBX family in DLBCL tissues and control group (non-tumor lymph nodes) [22]. According to the proportion of immune-reactive tumor cells, staining quantity could be divided into four levels: 0%, < 25%, 25–75%, and > 75%. The protein expression classification criteria based on staining intensity and staining quantity are as follows:negative, not detected; weak and < 25%, not detected; weak binding 25–75% or 75%, low; medium and < 25%, low; medium binding 25–75% or 75%, medium; strong and < 25%, medium; and strong combination 25–75% or 75%, strong.

### The association between the CBX family and patient prognosis

The online web database Genomicscape (http://genomicscape.com/) is a comprehensive tool that could be used to study the prognostic implications of genes in various cancers, which was established based on high-throughput data obtained from Gene Expression Omnibus (GEO) [23]. The prognostic value of the mRNA expression of CBX family in DLBCL patients was performed by Genomicscape (http://genomicscape.com/). The Significance Analysis of Microarrays (SAM) algo-rithms was used to determine the high/low expression groups (Wilcoxon test, FDR ≤ 0%, fold change ≥ 2, permutation = 300, unpaired). Kaplan–Meier survival plots with hazard ratio (HR), and log-rank *p*-value was shown on the webpage. *P*-value of < 0.05 was defined as the criterion for significance. Furthermore, multivariate COX regression analysis was performed with the Sanger-Box tool (http://www.sangerbox.com/home.html), which based on R package survival.

### The correlation between CBX family and immune cell infiltrate in DLBCL

Tumor Immune Estimation Resource 2.0 (TIMER2.0; http://timer.cistrome.org/) is an abundant web server for systematical analysis and visualization of immune infiltrates of various cancer types [24]. In our study, we utilized the ‘Gene’ module and the "correlation" module to estimated the correlation between CBX family gene expression and several tumor-associated immune cells as well as their immune markers. The correlation was expressed by the Spearman coefficient and was adjusted by purity.The red indicates a statistically significant positive association, and the blue indicates a statistically significant negative association. Gray denotes a non-significant result. *P*-value < 0.05 was considered statistically different.

### The relationship between CBX family and drug sensitivity in DLBCL cells

GSCALite (http://bioinfo.life.hust.edu.cn/web/GSCALite/) is a web-based platform for gene set cancer analysis, which is the dynamic analysis and visualization of gene sets in cancer pathway activity, methylation, and drug-sensitivity analysis [25]. The Spearman correlation was performed to detect the correlation between CBX family expression and 265 small molecules or drugs from Cancer Drug Sensitivity Genomics (GDSC). The positive correlation means the gene with high expression is resistant to the drug, vise verse. *P* < 0.05 was considered statistically significant. The critical value was a false discovery rate (FDR) of < 0.05.

### Confirmatory tests of CBX family protein expression in DLBCL

To further verify the protein expression level of CBX family in HPA database. The Clinical samples (18 paraffin DLBCL tissues and 18 non-tumor lymph nodes) were collected from the Department of Pathology, the First Affiliated Hospital of Jinan University. All patients provided informed consent for the study, which got the approval of Research Ethics Committee of Jinan University.

The collected tissue samples were fixed in a drying oven at 60° C for 30 min. After deparaffinization and rehydration treatment, the tissue slices were heated in a microwave oven with 1 × EDTA antigen retrieval solution at medium–high temperature for 30 min to retrieve antigens. Then, the slices were cooled naturally to room temperature. Next, the tissue sections were washed three times with PBS and incubated in 5% hydrogen peroxide for 10 min to inactivate endogenous peroxidase activity. Then, the tissues were blocked with goat serum (SL038, Solarbio, Beijing) for 30 min at room temperature. Subsequently, these slices were incubated with the primary CBX1 antibody (dilution: 1:400; ab10478, Abcam), CBX2 antibody (dilution: 1:200; ab235305, Abcam), CBX3 antibody (dilution: 1:2000; ab217999, Abcam), CBX5 antibody (dilution: 1:1000; ab109028, abcam), CBX6 antibody (dilution: 1:100; ab259848, abcam) at 4 °C overnight. The tissue slides were incubated with the secondary antibody (anti-rabbit Dako Envision + System HRP Labeled Polymer, Dako Ref#K4003) at room temperature for two hours. The tissue slices were washed with PBS and stained with 3,3' -diaminobenzidine solution for antigen detection (Dako Denmark A/S, Glostrup, Denmark). The slides were stained with DAB for 2 min and counterstained with hematoxylin to enhance the nuclear staining. Finally, the slides were installed, dehydrated by xylene and covered. At higher magnification (× 400), five visual fields were selected randomly, the expression positive signal was analyzed by ImageJ software. Compared the protein expression in DLBCL tissues and normal lymph node tissues according to the average optical density (AOD) as a parameter for semi-quantitative detection.

### Statistical analysis

All statistical analyses were processed using SPSS statistical software (version 25.0; IBM, Armonk, NY, USA). Kaplan–Meier and multivariate Cox regression methods were used to analyze the prognosis of DLBCL patients. The CBX1/2/3/5/6 protein expression levels between the cancer group and the control group were compared by Student’s-t test. The data in this study were presented as mean ± SD. *P* < 0.05 was considered statistically significant.

## Results

### The mRNA expression levels of CBX family in DLBCL patients

**Fig. 2:**
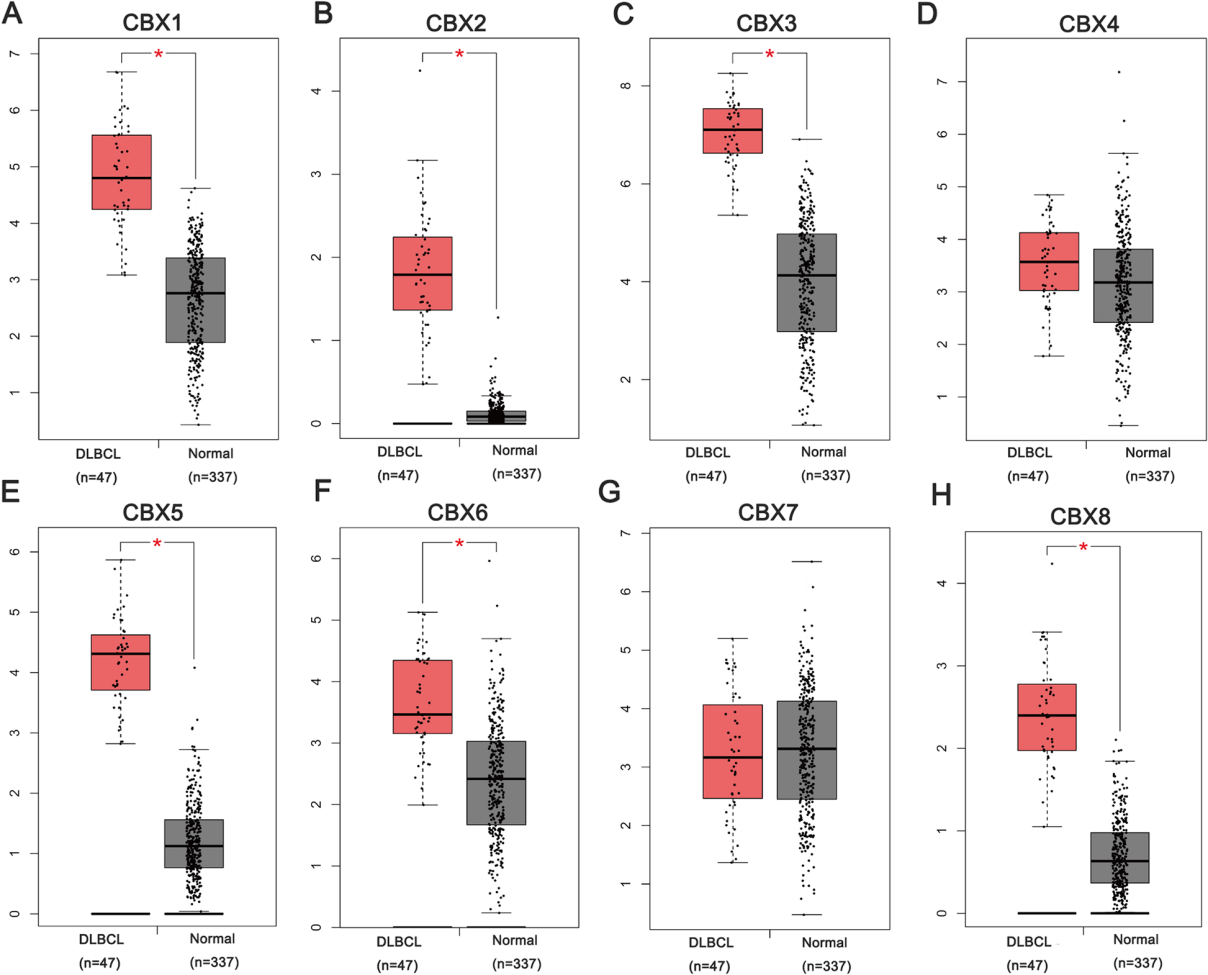
The mRNA expression levels of CBX family in Diffuse large B cell lymphoma (DLBCL) (GEPIA). **A** CBX1. **B** CBX2. **C** CBX3. **D** CBX4. **E** CBX5. **F** CBX6. **G** CBX7. **H** CBX8. *P < 0.05

To explore the expression differences of the CBX family in DLBCL patients, the GEPIA database was used (Fig. 2). Based on the data obtained from GEPIA, the expression levels of CBX1, CBX2, CBX3, CBX5, CBX6, and CBX8 in DLBCL were higher than normal samples. However, CBX4 and CBX7 did not differ significantly.

In the Oncomine dataset, the transcriptional levels of CBX1, CBX2, CBX3, CBX5, CBX6, and CBX8 were significantly elevated in DLBCL vs. normal samples, while the transcriptional levels of CBX4 and CBX7 were not statistically significant (Table 1). These results were consistent with data from GEPIA. Then, the CBX family members were selected for further analysis (excepting for CBX4, CBX7).

**Table 1:** Differential expression analyses of CBX family in DLBCL (Oncomine)

Genes	Type	cases	*P*-Value	Fold Change	T-test	Reference
CBX1	DLBCL vs. normal	336	3.80E-02	1.320	1.807	Basso lymphoma [26]
	DLBCL vs. normal	67	1.89E-04	1.628	4.215	Brune lymphoma [27]
CBX2	DLBCL vs. normal	336	1.00E-03	1.419	3.747	Brune lymphoma [27]
	DLBCL vs. normal	136	0.00E-03	1.101	2.835	Campagno lymphoma [28]
CBX3	DLBCL vs. normal	67	3.70E-04	1.470	4.032	Brune lymphoma [27]
	DLBCL vs. normal	27	2.70E-02	1.462	2.245	Storz lymphoma [29]
	DLBCL vs. normal	336	2.40E-02	1.314	2.201	Basso lymphoma [26]
CBX5	DLBCL vs. normal	67	4.91E-04	2.131	4.485	Brune lymphoma [27]
	DLBCL vs. normal	136	3.23E-05	1.463	4.325	Campagno lymphoma [28]
	DLBCL vs. normal	27	1.00E-02	2.173	2.828	Storz lymphoma [29]
CBX6	DLBCL vs. normal	136	1.50E-06	1.523	5.443	Campagno lymphoma [28]
	DLBCL vs. normal	67	2.63E-05	1.395	5.976	Brune lymphoma [27]
	DLBCL vs. normal	27	2.00E-03	2.676	3.600	Storz lymphoma [29]
CBX8	DLBCL vs. normal	136	7.67E-07	1.252	5.434	Campagno lymphoma [28]
	DLBCL vs. normal	67	2.40E-02	1.062	2.052	Brune lymphoma [27]

A mass of cancer cell lines in CCLE could provide reliable guidance on the gene expression in cancer subtypes of different tissue origins. By assembling CCLE, it was clear that CBX1, CBX2, CBX3, CBX5, CBX6, and CBX8 were all highly expressed in DLBCL cell lines. Meanwhile, the relative expression levels of the 6 genes were detected in 18 common DLBCL cell lines. The details were in Figure S1.

### Co-expression, interaction, and functional enrichment analyses of the CBX family

**Fig. 3:**
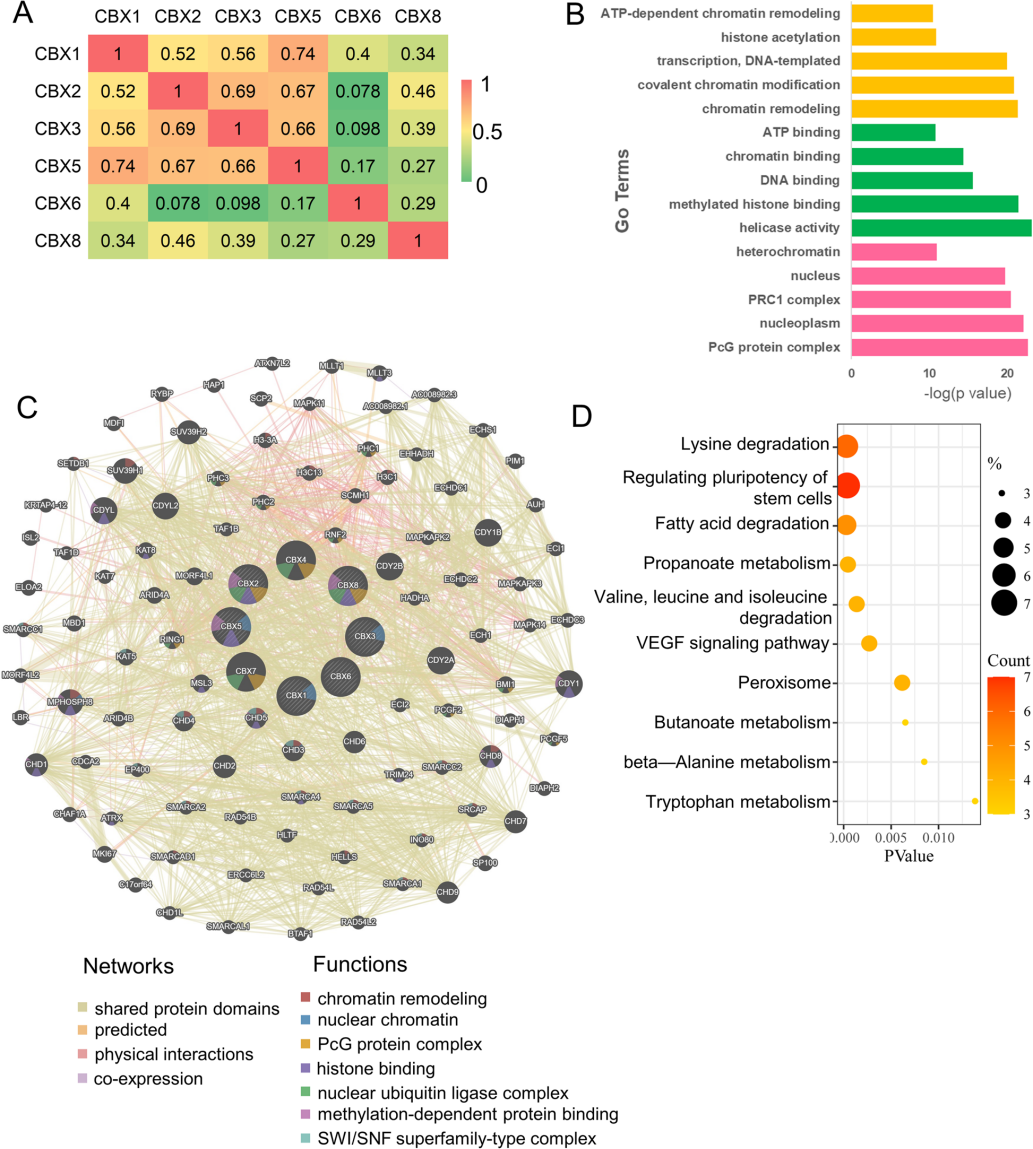
Co-expression and interaction analyses of CBX family members at the gene and protein levels. **A** Pearson correlation of CBX family members (GEPIA). The numbers in the color block represent their correlation coefficients. **B** Network for CBX family protein and the 100 most frequently altered neighbor genes (GeneMANIA). Each node represented a gene, and the size of the node represented the strength of gene interaction. The color of the nodes indicated the possible function of each gene, and the color of the connecting line between the nodes represented the type of gene–gene interaction. **C** The Gene Ontology (GO) enrichment analysis: the top 5 terms of biological process (BP), molecular function (MF), and cellular component (CC) were shown. The yellow bar chart, BP; The green bar chart, MF; The red bar chart, CC. **D** The top 10 Kyoto Encyclopedia of Genes and Genomes (KEGG) enrichment analysis. *P* < 0.05 was considered to indicate a statistically signifificant difference. The pathways shown in Fig. 3D were obtained from the KEGG database [30–32]

Next, we conducted bioinformatics analysis to explore the interaction and co-expression of the differentially expressed CBX family in DLBCL (including CBX1, CBX2, CBX3, CXB5, CBX6, and CBX8). Pearson’s test was used to assess the correlation between the genes, and the results showed a strong correlation between CBX1 and CBX5 (Fig. 3A). Moreover, the functional network diagram of the CBX family and the 100 most frequently altered adjacent genes were executed by the GeneMANIA database (Fig. 3B). The 6 central nodes of CBX family were surrounded by 100 nodes. These nodes represented genes that were closely related to CBX family members in sharing protein domains, prediction, physical interactions, and co-expression. In addition, the functions of these 106 genes were mainly related to chromatin remodeling, nuclear chromatin, PcG protein complex, histone binding, nuclear ubiquitin ligase complex, methylation-dependent protein binding, and SWI/SNF superfamily-type complex.

Then, KEGG and GO enrichment analyses were conducted to further investigate the potential biological functions of the 106 interactive genes using the DAVID 6.8 database. The GO enrichment analysis results were shown in Fig. 3C. GO describes the genes in three ways, namely BP, MF, and CC. In the top 5 BP group, the genes were primarily enriched in “ATP-dependent chromatin remodeling”, “histone acetylation”, “transcription, DNA-templated”, “covalent chromatin modification”, and “chromatin remodeling”. In the top 5 MF group, the genes were mainly enriched in “ATP binding”, “chromatin binding”, “DNA binding”, “methylated histone binding”, and “helicase activity”. In the top 5 CC group, the genes were principally enriched in “heterochromatin”, “nucleus”, “PRC1 complex”, “nucleoplasm”, and “PcG protein complex”. Among the top 10 KEGG pathways, the main pathways involved were “Lysine degradation”, “Signaling pathways regulating pluripotency of stem cells”, “VEGF signaling pathway”, which may be participated in the tumorigenesis of DLBCL (Fig. 3D).

### The protein expression levels of CBX family in DLBCL tissues

**Fig. 4:**
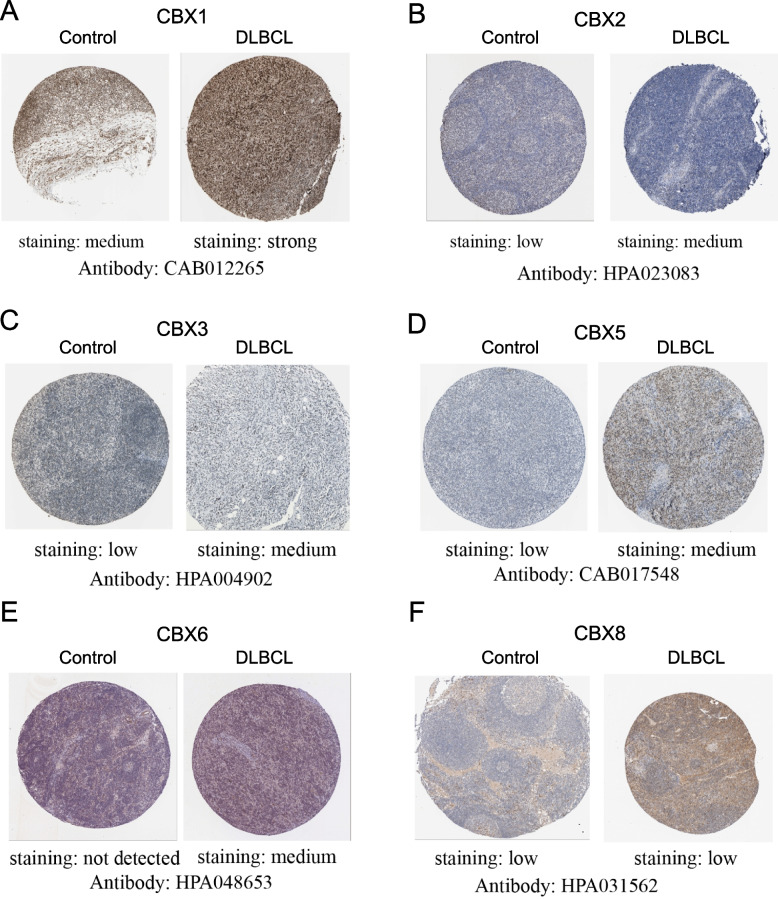
Representative immunohistochemistry staining images in control groups and DLBCL tissues from the Human Protein Atlas (HPA) database. **A** CBX1. **B** CBX2. **C** CBX3. **D** CBX5. **E** CBX6. **F** CBX8

To further explored the protein expressions of CBX family in DLBCL, we explored immunohistochemistry staining images from the HPA database. The results showed that the protein levels of CBX1, CBX2, CBX3, CBX5, and CBX6 were significantly higher in the DLBCL tissues when compared to control groups, except for the low expression level of CBX8 in control groups and DLBCL tissues (Fig. 4).

### The prognostic value of mRNA expression of the CBX family in DLBCL patients

**Fig. 5:**
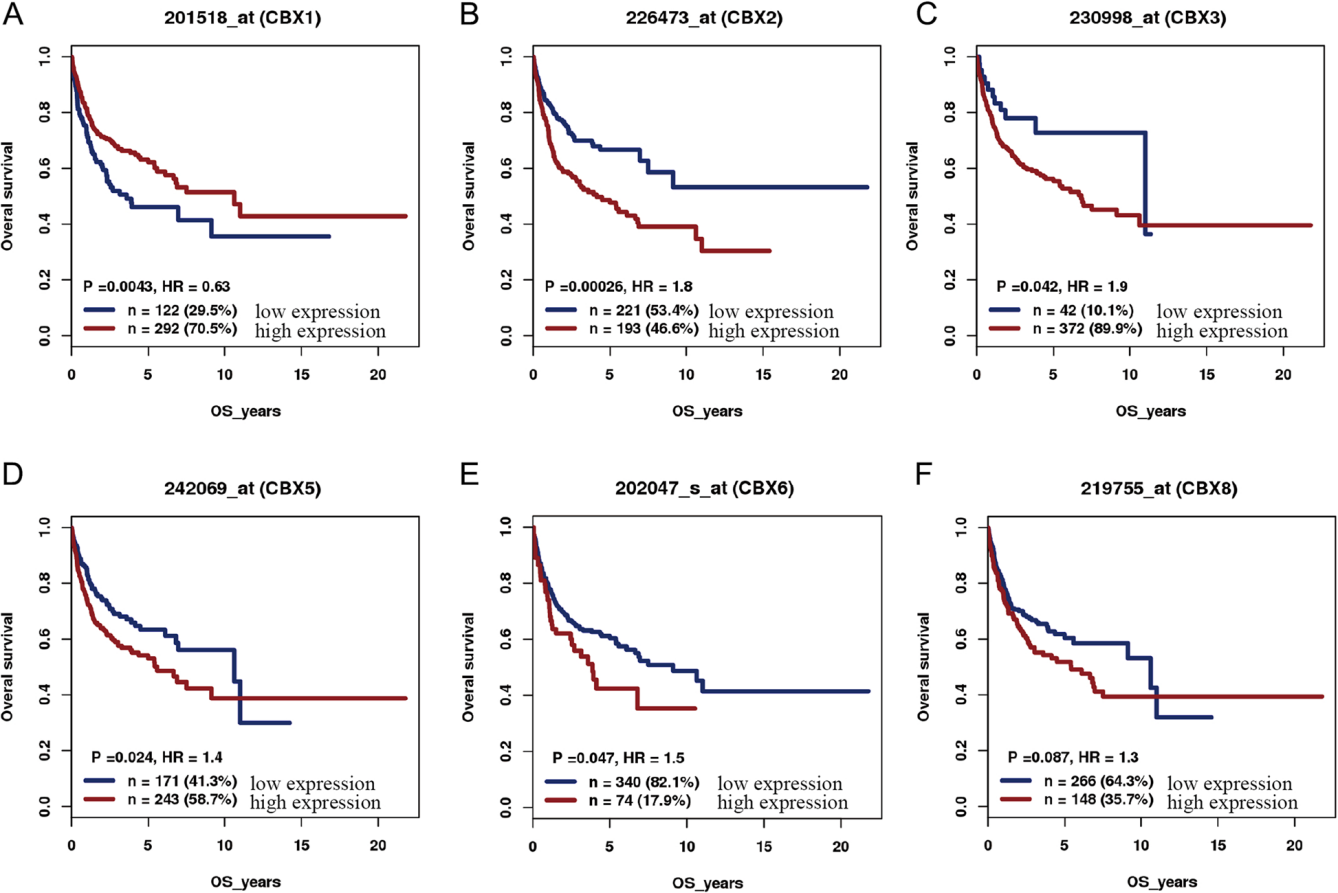
Kaplan–Meier curve for overall survival (OS) of DLBCL in GenomicScape. **A** CBX1. **B** CBX2. **C** CBX3. **D** CBX5. **E** CBX6. **F** CBX8. The *P*-values are calculated using log-rank statistics. *P* < 0.05 was considered statistically significant; HR, hazard ratio

To evaluated the relationship between the mRNA expressions of CBX family and clinical outcomes in DLBCL patients, Kaplan-Meier survival analysis was conducted with data from GSE10846 and visualized with GenomicScape online analysis tool. As shown in Fig. 5, the high expressions of the CBX family (including CBX2, CBX3, CBX5, and CBX6) in tumor tissues were related to the worse OS of DLBCL patients, whereas high expression of CBX1 was correlated with better prognosis of patients.

**Fig. 6:**
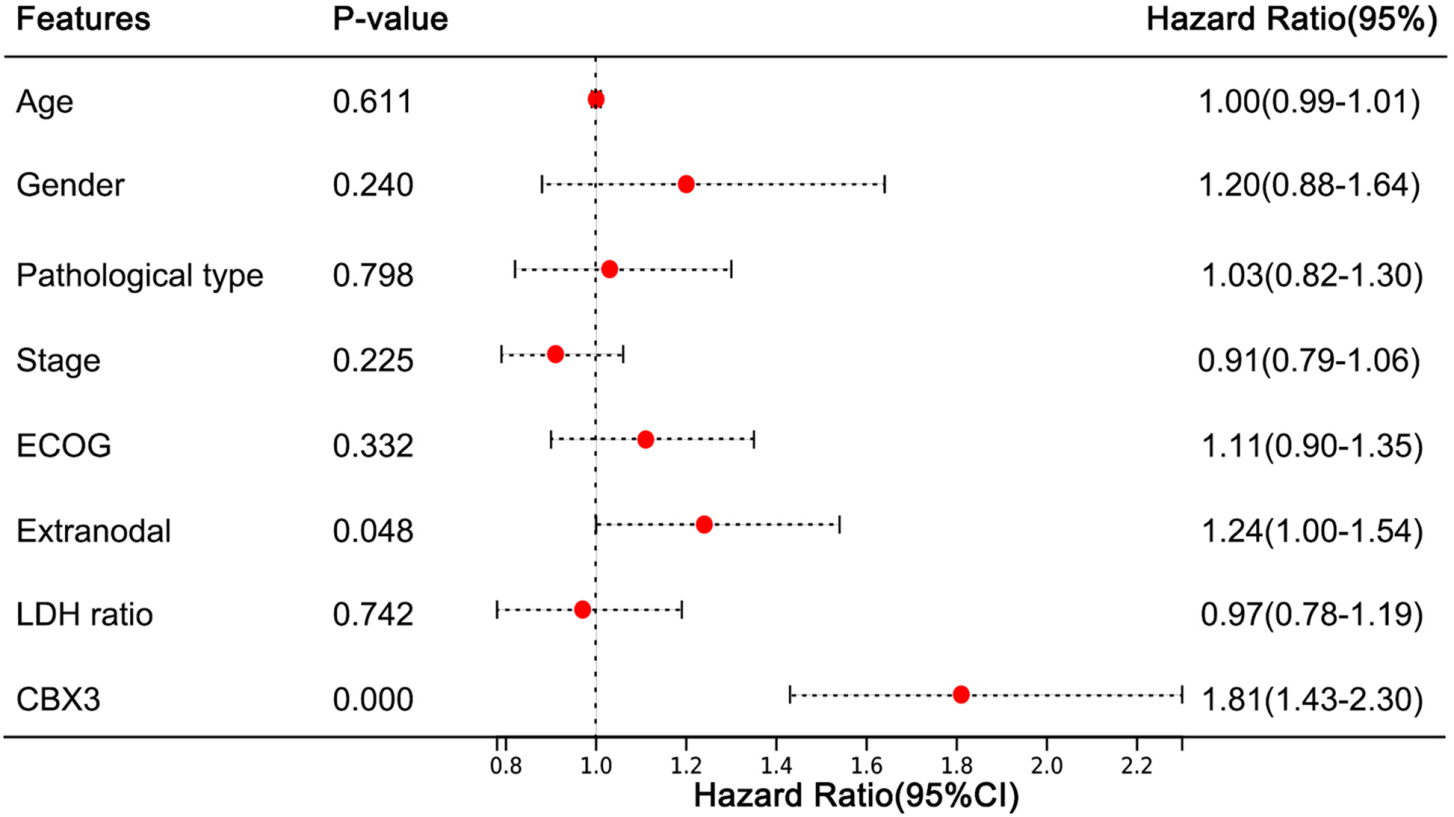
Forest plot of the multivariate Cox regression analysis of CBX3 in Diffuse Large B-cell Lymphoma (DLBCL). The threshold *P*-value was defined as 0.05

Furthermore, whether the CBX family could be used as independent prognostic predictors were under investigation by multivariate cox regression. As shown in Fig. 6, consistent with the Kaplan-Meier survival analysis above, CBX3 (HR = 1.81, 95%CI = 1.43-2.30, p = 0.000) was identified as independent prognostic predictor. The results of CBX1, CBX2, CBX5, CBX6, and CBX8 were shown in Figure S2.

### Correlations between CBX family and immune cell infiltration in DLBCL

**Fig. 7:**
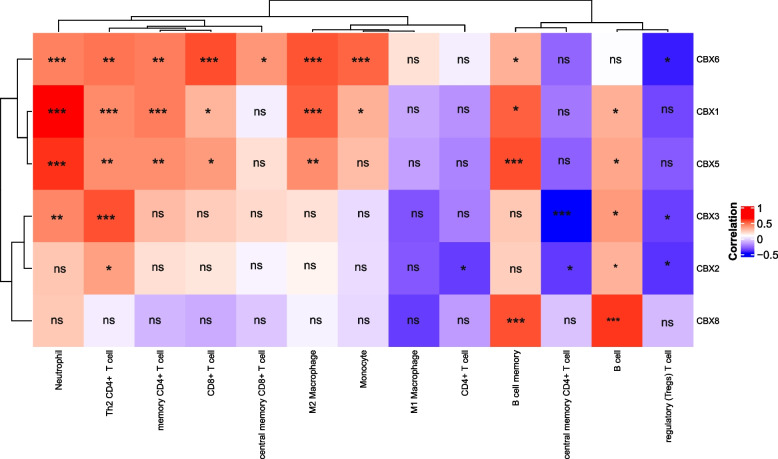
Correlation heatmap between the expression level of CBX family and the immune cell infiltration. The number shows the correlation value. ns, no statistical significance; *p < 0.05; **p < 0.01; ***p < 0.001

Considering that the level of immune cell infiltration is related to the proliferation and progression of cancer cells, it could independently predict the survival rate and lymph node metastasis of cancer patients [33–35]. As such, we embarked on a comprehensive investigation on the relationship between CBX family and immune cell infiltration in DLBCL using TIMER 2.0, and correlation was adjusted by tumor purity (Fig. 7). We found that the expressions of CBX1, CBX5, and CBX6 were in positive connection with the infiltration of memory B cells, CD4 + memory T cells, CD4 + Th2 T cells, CD8 + T cells, neutrophils, and M2 macrophages. In addition, CBX1 was positively correlated with monocytes and B cells infiltration, CBX5 was positively correlated with B cells infiltration, and CBX6 was correlated with CD8 + central memory T cells, monocytes infiltration, and regulatory T cells (Tregs). CBX2 expression was positively associated with the infiltration of B cells and CD4 + Th2 T cells, while was negatively associated with the infiltration of CD4 + central memory T cells, CD4 + T cells, and Tregs. Similarly, the expression of CBX3 was positively associated with the infiltration of B cells, CD4 + Th2 T cells, and neutrophils, but was negatively associated with the infiltration of CD4 + central memory T cells and Tregs. There was a positive relationship between CBX8 expression and the infiltration of B cells as well as memory B cells. In summary, CBX family may play an important role in immune cell infiltration of DLBCL, especially CBX1, CBX5, and CBX6.

### Assessment of the relationship between CBX family and immune checkpoints

The anti-cancer effect of immune checkpoint inhibitors not only requires the abundance of lymphocyte infiltration in the TME, but also relies on the high expression level of immune checkpoints of tumor cells. We further explored the link between CBX family and various immune markers for different immune cells in DLBCL through the TIMER 2.0 database, including monocytes, tumor-associated macrophages (TAMs), macrophages, neutrophils, NK cell, and dendritic cells, etc. After adjusting these results based on tumor purity, the expression levels of CBX1, CBX5, and CBX6 were significantly related to most of the immune markers of DLBCL tumor-infiltrating immune cells, and the results were consistent with the analysis of immune cell infiltration (Table 2). The role of Tex/deplete NK cell immune checkpoints in tumors is a hot topic of current research. Interestingly, we found that the increased expression of CBX1, CBX5, and CBX6 strongly correlated with high expression of PDL1(CD274), CD155 (PVR), PVRL3(CD113), CD226(DNAM-1), and CD96 in DLBCL.

**Table 2:** Correlation analysis between the CBX family and related markers of immune cells

Immune cells	Immune checkpoint	Purity		CBX1		CBX2		CBX3		CBX5		CBX6		CBX8	
		cor	p	cor	p	cor	P	Cor	P	Cor	P	Cor	P	Cor	P
CD8 + T cell	CD8A	0.544	***	0.332	*	0.291	0.065	0.399	**	0.427	**	0.580	***	0.087	0.589
	CD8B	0.411	**	0.055	0.733	0.045	0.779	0.147	0.359	0.239	0.132	0.229	0.151	0.094	0.561
T cell (general)	CD3D	-.709	***	0.116	0.472	0.174	0.276	0.100	0.533	0.095	0.555	0.034	0.832	0.102	0.524
	CD3E	0.750	***	0.472	0.664	0.276	0.483	0.533	0.699	0.555	0.704	0.832	0.874	0.524	0.695
	CD2	0.737	***	0.121	0.452	0.011	0.946	0.114	0.480	0.172	0.281	0.384	0.013	0.045	0.779
B cell	CD19	0.145	0.361	0.036	0.823	0.019	0.908	0.069	0.667	0.069	0.669	0.305	0.053	0.087	0.589
	CD79A	0.032	0.841	0.189	0.236	0.283	0.073	0.346	*	0.246	0.121	0.017	0.916	0.529	***
Monocyte	CD86	0.385	*	0.465	**	0.067	0.678	0.141	0.379	0.424	**	0.491	**	0.162	0.312
	CD115 (CSF1R)	0.514	***	0.483	**	0.147	0.360	0.202	0.205	0.449	**	0.590	***	0.070	0.664
TAM	CCL2	0.252	0.107	0.383	*	0.001	0.996	0.015	0.927	0.221	0.164	0.303	0.054	0.121	0.451
	CD68	0.410	**	0.196	0.219	0.036	0.823	0.020	0.901	0.171	0.286	0.490	0.001	0.076	0.635
	IL10	0.211	0.180	0.466	**	0.239	0.133	0.345	*	0.403	**	0.563	***	0.050	0.758
M1 Macrophage	INOS (NOS2)	0.195	0.216	0.252	0.112	0.084	0.602	0.073	0.651	0.171	0.286	0.286	0.070	0.137	0.392
	IRF5	0.257	0.100	0.442	**	0.108	0.503	0.084	0.601	0.375	*	0.425	**	0.196	0.218
	COX2 (PTGS2)	0.324	*	0.434	**	0.159	0.319	0.223	0.161	0.438	**	0.432	**	0.027	0.868
M2 Macrophage	CD163	0.084	0.597	0.254	0.109	0.054	0.737	0.098	0.541	0.156	0.330	0.489	**	0.191	0.231
	VSIG4	0.157	0.319	0.121	0.452	0.063	0.696	0.018	0.910	0.049	0.760	0.415	**	0.237	0.135
	MS4A4A	2.202	0.200	0.290	0.066	0.049	0.762	0.050	0.755	0.115	0.476	0.291	0.065	0.229	0.150
Neutrophils	CD66b(CEACAM8)	0.273	0.080	0.086	0.592	0.214	0.179	0.227	0.153	0.101	0.529	0.001	0.996	0.197	0.216
	CD11b (ITGAM)	0.309	*	0.260	0.101	0.082	0.610	0.100	0.535	0.257	0.104	0.337	*	0.234	0.140
	CCR7	0.498	***	0.268	0.090	0.047	0.769	0.157	0.327	0.222	0.163	0.288	0.068	0.049	0.759
NK cell	KIR2DL1	0.352	*	0.107	0.506	0.022	0.891	0.010	0.951	0.082	0.610	0.114	0.476	0.040	0.805
	KIR2DL3	0.424	**	0.158	0.324	0.074	0.647	0.023	0.885	0.226	0.156	0.092	0.569	0.070	0.663
	KIR2DL4	0.206	0.191	0.237	0.135	0.034	0.834	0.052	0.747	0.135	0.400	0.222	0.162	0.209	0.190
	KIR3DL1	0.285	0.067	0.133	0.406	0.131	0.416	0.019	0.908	0.159	0.322	0.199	0.212	0.010	0.951
	KIR3DL2	0.612	***	0.281	0.075	0.307	0.051	0.163	0.307	0.301	0.056	0.268	0.090	0.017	0.916
	KIR3DL3	0.117	0.461	0.091	0.572	0.111	0.490	0.004	0.979	0.053	0.743	0.072	0.656	0.149	0.354
	KIR2DS4	0.239	0.127	0.163	0.309	0.305	0.052	0.252	0.112	0.175	0.274	0.227	0.154	0.152	0.343
Dendritic cell	HLA-DPB1	0.207	0.188	0.335	*	0.186	0.245	0.157	0.327	0.223	0.162	0.170	0.288	0.072	0.656
	HLA-DQB1	0.160	0.311	0.070	0.664	0.002	0.989	0.091	0.570	0.076	0.639	0.031	0.850	0.094	0.559
	HLA-DRA	0.195	0.215	0.119	0.458	0.131	0.416	0.142	0.377	0.126	0.432	0.151	0.345	0.299	0.058
	HLA-DPA1	0.304	*	0.134	0.404	0.070	0.664	0.138	0.388	0.181	0.258	0.232	0.144	0.170	0.288
	BCDA-1 (CD1C)	0.026	0.872	0.151	0.348	0.365	*	0.457	**	0.268	0.090	0.113	0.480	0.499	***
	BDCA-4 (NRP1)	-.263	0.092	0.591	0.000	0.352	*	0.276	0.080	0.518	***	0.637	***	0.054	0.737
	CD11c (ITGAX)	0.533	***	0.311	*	0.083	0.605	0.049	0.763	0.275	0.082	0.242	0.127	0.400	**
Th1	TBX21	0.706	***	0.178	0.265	0.055	0.732	0.017	0.918	0.135	0.401	0.353	*	0.242	0.128
	STAT4	0.732	***	0.315	*	0.005	0.977	0.091	0.572	0.242	0.128	0.310	*	0.099	0.538
	STAT1	0.451	**	0.493	**	0.214	0.180	0.242	0.128	0.444	**	0.755	***	0.117	0.467
	IFN-g (IFNG)	0.537	***	0.401	**	0.231	0.146	0.301	0.056	0.347	*	0.665	***	0.009	0.957
	TNF-a (TNF)	0.236	*	0.242	0.128	0.223	0.160	0.122	0.446	0.386	*	0.383	*	0.060	0.711
Th2	GATA3	0.688	***	0.273	0.084	0.110	0.493	0.118	0.462	0.314	*	0.417	**	0.102	0.526
	STAT6	0.065	0.684	0.859	***	0.500	***	0.567	***	0.847	***	0.693	***	0.327	*
	STAT5A	0.418	***	0.101	0.531	0.116	0.469	0.214	0.179	0.046	0.775	0.069	0.666	0.144	0.370
	IL13	0.298	0.055	0.108	0.501	0.189	0.237	0.211	0.186	0.047	0.772	0.063	0.693	0.020	0.899
Tfh	BCL6	0.181	0.251	0.429	0.005	0.294	0.062	0.412	0.007	0.328	0.037	0.177	0.269	0.303	0.054
	IL21	0.411	**	0.356	*	0.021	0.897	0.022	0.893	0.313	*	0.435	**	0.160	0.317
Th17	STAT3	0.277	0.076	0.706	***	0.411	**	0.479	**	0.693	***	0.721	***	0.144	0.368
	IL17A	0.508	***	0.110	0.494	0.139	0.386	0.027	0.869	0.063	0.695	0.141	0.378	0.054	0.735
Treg	FOXP3	0.633	***	0.313	*	0.037	0.819	0.033	0.836	0.303	0.054	0.307	0.051	0.355	*
	CCR8	0.477	**	0.469	**	0.222	0.164	0.228	0.152	0.468	**	0.521	***	0.428	**
	STAT5B	0.323	0.037	0.639	***	0.408	**	0.435	**	0.648	***	0.722	***	0.360	*
	TGFb (TGFB1)	0.517	***	0.628	***	0.468	**	0.328	*	0.527	***	0.723	***	0.417	**
Tex	PD-1 (PDCD1)	0.533	***	0.115	0.473	0.256	0.107	0.397	*	0.226	0.155	0.065	0.687	0.201	0.208
	CTLA4	0.702	***	0.006	0.971	0.137	0.391	0.021	0.895	0.095	0.553	0.180	0.261	0.030	0.854
	LAG3	0.560	***	0.041	0.797	0.037	0.819	0.078	0.630	0.078	0.629	0.315	0.045	0.198	0.214
	TIM-3 (HAVCR2)	0.387	*	0.340	*	0.061	0.704	0.187	0.242	0.288	0.068	0.551	***	0.082	0.609
	GZMB	0.243	0.122	0.136	0.396	0.084	0.600	0.125	0.437	0.100	0.533	0.459	**	0.216	0.174
	PDL1 (CD274)	0.365	*	0.636	***	0.383	*	0.372	*	0.606	***	0.864	***	0.156	0.330
	TIGIT	0.432	**	0.161	0.314	0.042	0.796	0.071	0.661	0.141	0.380	0.198	0.215	0.153	0.341
	CD112 (PVRL2)	0.432	**	0.037	0.818	0.115	0.476	0.202	0.205	0.045	0.782	0.263	0.097	0.171	0.286
	CD155 (PVR)	0.436	***	0.363	*	0.139	0.384	0.174	0.277	0.363	*	0.626	***	0.120	0.454
	CD113 (PVRL3)	0.508	***	0.655	***	0.424	**	0.479	**	0.613	***	0.674	***	0.486	**
	CD226 (DNAM-1)	0.662	***	0.619	***	0.260	0.101	0.440	**	0.612	***	0.601	***	0.314	*
	CD96	0.704	***	0.407	**	0.145	0.365	0.223	0.160	0.306	*	0.440	**	0.102	0.525

^*^p<0.05, **p<0.01, ***p<0.001

### The relationship between CBX family and drug sensitivity in DLBCL cells

**Fig. 8:**
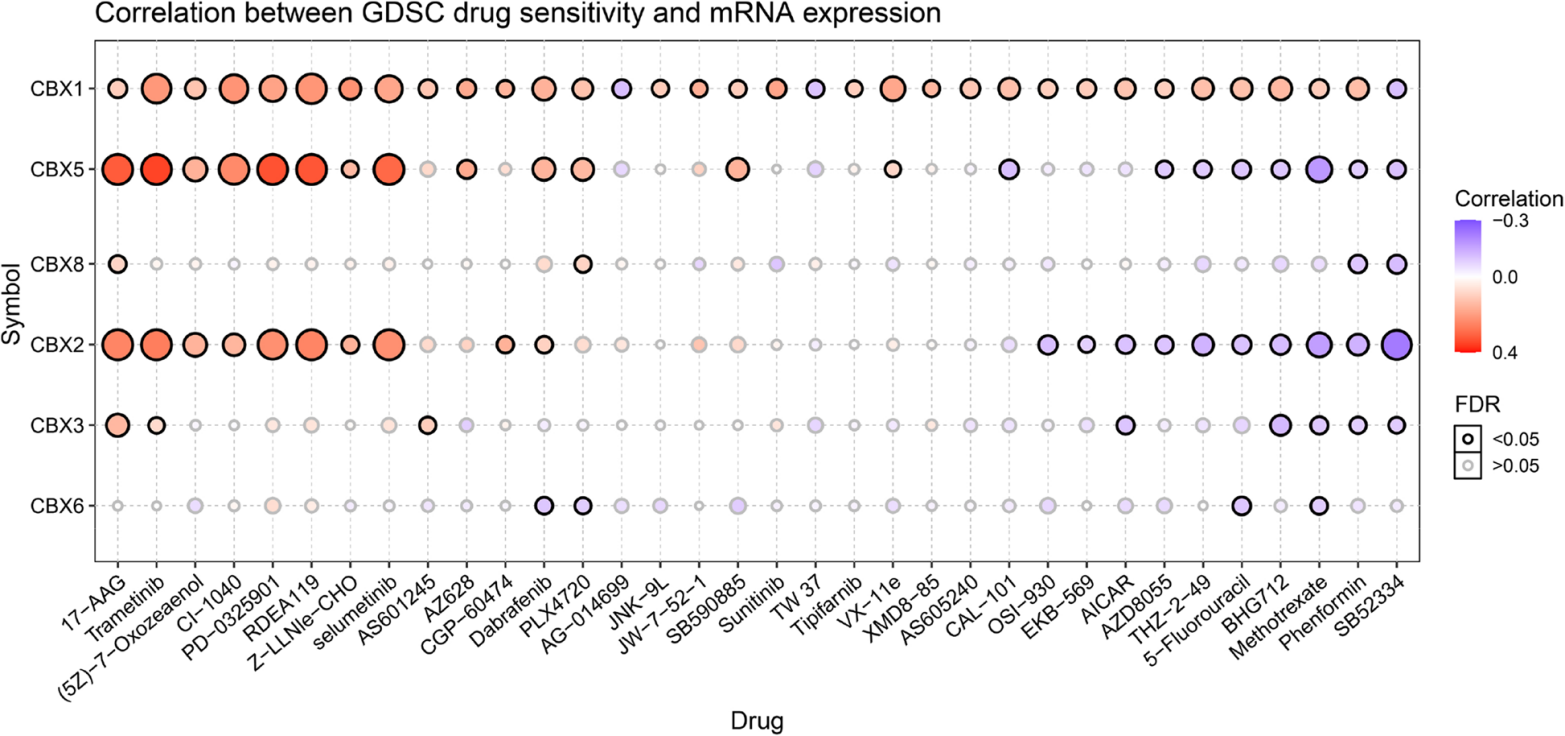
Drug sensitivity analysis of CBX family in Diffuse Large B-cell Lymphoma (DLBCL) cells (GSCALite). The Spearman correlation represents the relationship between gene expression and drug. The positive correlation means that the gene high expression is resistant to the drug, vise verse. The critical value was a false discovery rate (FDR) of < 0.05

We next explored the correlation between the expression of CBX family in DLBCL cells and small-molecule drugs sensitivity using the GDSC IC50 drug data from the GSCALite database. Drug sensitivity revealed that DLBCL cell lines with CBX1/2/3/5/6/8-overexpression were sensitive to certain small molecule drugs, including target drugs and non-target drugs (Fig. 8). And we found that DLBCL cells with high CBX1 expression were resistant to most of the drugs in the figure. However, CBX2/5 had two polarities. In addition, high expression of CBX6/8 did not affect the sensitivity of DLBCL to drugs. Details of common small-molecule drugs were presented in Table S1.

### Verification in DLBCL tissues by immunohistochemistry

**Fig. 9:**
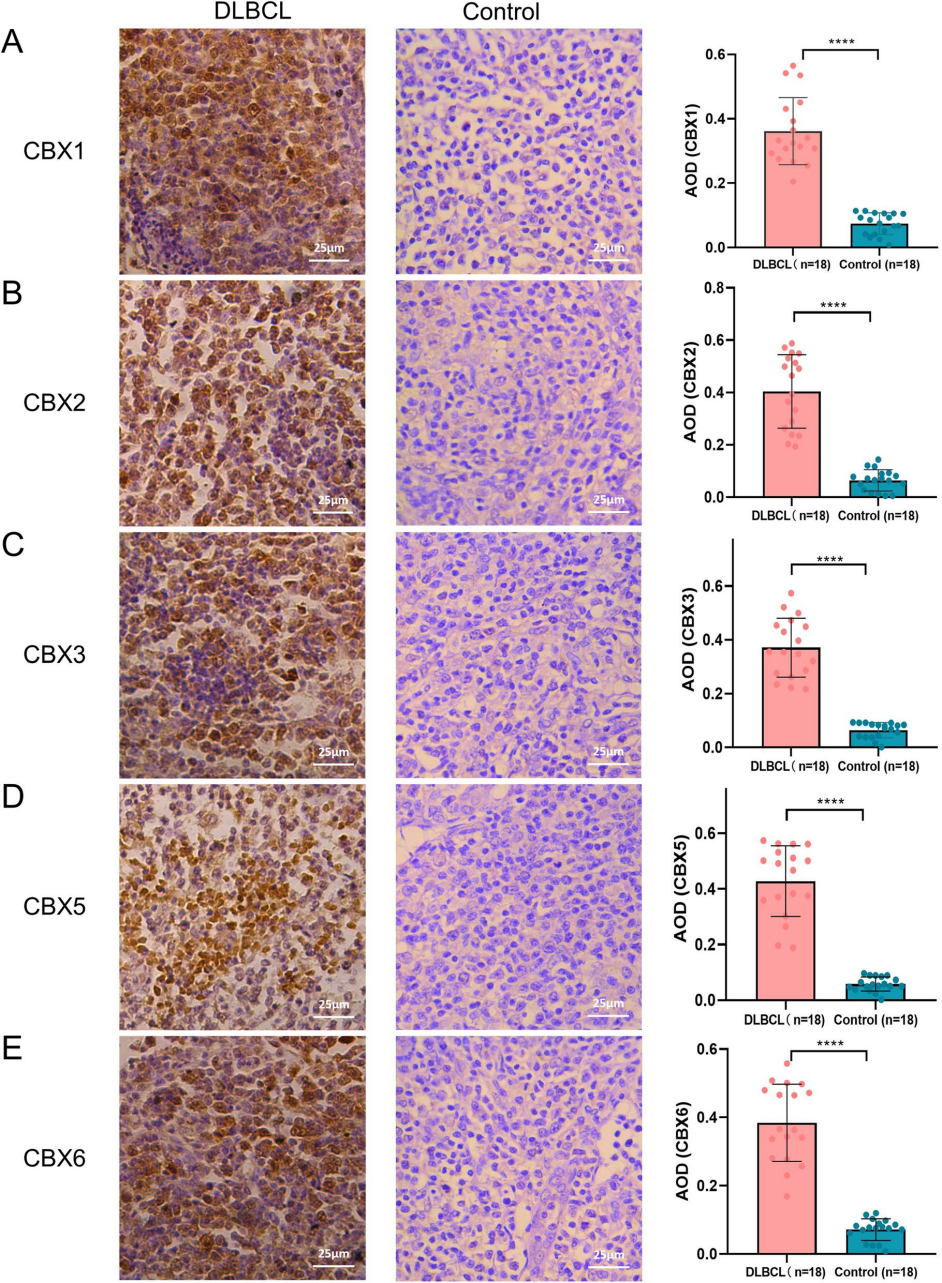
The expression levels of CBX1, CBX2, CBX3, CBX5, and CBX6 in Diffuse Large B-cell Lymphoma (DLBCL) tissues and control groups. **A**-**E** The expression level of CBX1(**A**), CBX2(**B**), CBX3(**C**), CBX5(**D**), and CBX6(**E**) in DLBCL tissues and control groups (× 400). **F** The average optical density (AOD) of staining in each tissue slide. Values are expressed as the mean ± SD. ****P < 0.0001

To verified the protein expressions of CBX family in DLBCL tissues, immunohistochemistry was performed to detected the protein expressions of CBX1/2/3/5/6 in DLBCL tissues and control groups (non-tumor lymph nodes). As shown in Fig. 9, CBX1, CBX2, CBX3 , CBX5 and CBX6 immunoreactivity were observed in the nucleus and yellowish brown cells were recognized as positive. CBX1 protein expression was obviously elevated in DLBCL specimens (0.351 ± 0.098) compared with control groups (0.074 ± 0.034, *P* < 0.0001). Similar results were also obtained for CBX2 (DLBCL specimens: 0.351 ± 0.098; control groups: 0.065 ± 0.041, *P* < 0.0001), CBX3 (DLBCL specimens: 0.374 ± 0.112; control groups: 0.064 ± 0.028, *P* < 0.0001) , CBX5 ( DLBCL specimens: 0.420 ± 0.127; control groups: 0.058 ± 0.025, *P* < 0.0001) and CBX6 ( DLBCL specimens: 0.379 ± 0.115; control groups: 0.072 ± 0.032, *P* < 0.0001) protein expression. In summary, our results showed that the CBX1, CBX2, CBX3, CBX5 and CBX6 proteins were overexpressed in DLBCL tissues compared with control groups. There were consistent with the results in the HPA database.

## Discussion

DLBCL is highly aggressive with rapid disease progression, accounting for more than 80% of invasive lymphoma cases in the world [36]. Earlier diagnosis means better prognosis for high-risk DLBCL patients. As an indispensable part of the polycomb group complex, CBX family has been shown to play an important role in various solid tumors, such as breast cancer, liver cancer, lung cancer, etc. [7, 8, 10], but the biological function and the prognostic value of CBX family in DLBCL are unclear. We first analyzed the expressions of CBX family between DLBCL tissues and normal tissues at the mRNA and protein level using different databases, and we performed immunohistochemistry to validate our analysis. Then, we analyzed the association between the expression levels of CBX family and patient prognosis, and performed multifactorial COX regression survival analysis to construct prediction models. Then, we investigated the relationship between CBX family and immune infiltration in DLBCL patients. Finally, we tried to find sensitive drugs in DLBCL cell lines with high CBX family expression by drug sensitivity analysis, which may be a potential therapeutic modality. We found that: 1) The mRNA and protein expressions of CBX1/2/3/5/6 were higher in DLBCL patient tissues than control groups, and immunohistochemistry validated our results. 2) The high expressions of CBX2/3/5/6 were associated with worser OS in DLBCL patients, and multivariate COX regression analysis showed that the expression level of CBX3 could predict the patients prognosis together with other clinical variables (e.g., age, gender, pathological type, ECOG score, extranodal metastasis, LDH ratio) . 3) In DLBCL tissues, the expressions of CBX1/5/6 were significantly correlated with immune cell infiltration in the tumor microenvironment. 4) DLCBL cell lines with CBX family over-expression were sensitive to a number of small molecule drugs that had the potential to become therapeutic agents.

We first explored the differential expression levels of the CBX family in DLBCL. We found that most members of CBX family were abnormally highly expressed in DLBCL (except CBX4 and CBX7) compared with control groups. Our research results showed that CBX1/2/3/5/6 significantly over-expressed at the transcriptome levels and protein levels both in the DLBCL cells and DLBCL tissues. The results of immunohistochemistry validated this data analysis. Interestingly, CBX8 only increased at the transcriptome level, the protein expression of CBX8 in DLBCL tumor tissues and control groups had no significant difference. Previous studies showed that the CBX family was critically involved in the regulation of various biological functions, such as gene expression and physical development [37]. Yan et al. [38] found that LINC00857 contributed to DLBCL proliferation and lymphomagenesis through regulating miR-370-3p/CBX3 axis. CBX2 played a crucial role in leukemia progression and highlighted the potential drug role of the CBX2-P38 MAPK network in AML [39]. Our conclusion was helpful for further research on the role of CBX family in malignant tumors, especially hematological tumors.

In our study, Kaplan–Meier survival analysis showed that the high mRNA expressions of CBX2/3/5/6 were associated with poor prognosis of DLBCL patients, whereas high expression of CBX1 was correlated with better prognosis of patients. Through literature review and analysis, we have found that the role of CBX1 in tumor development is not clear. Previous research indicated that high expression of CBX1 was associated with worse clinical outcomes in Hepatocellular Carcinoma [40]. Conversely, in Clear cell renal cell carcinoma patients, the Kaplan–Meier curve analysis revealed that high expression of CBX1 was significantly correlated with better OS and disease-free survival [41]. This intriguing finding highlights the importance of further investigation. Consistent with Kaplan–Meier analysis, CBX3 had a higher hazard ratio in multivariate regression analysis, suggesting that CBX3 may be associated with poor prognosis and identified as an independent prognostic marker. Previous studies suggested that the high expressions of CBX family were associated with the prognosis of patients with various solid tumors. Such as, Zhou et al. [42] found that CBX2 may function as an oncogene and potential prognostic biomarker in colorectal cancer; CBX3 protein expression was increased in prostate cancer, and Cox survival analysis showed that it was an independent prognostic predictor [43]. Therefore, we hypothesized that CBX family as a potential prognostic marker may be associated with poorer prognosis in DLBCL patients.

Immune cells in TME have been demonstrated to have the activity of promoting or suppressing tumors. They were considered significant determinants of patient clinical outcome and immunotherapy response [44–46]. In our research, the mRNA expressions of CBX family (especially CBX1, CBX5, and CBX6) in DLBCL were significantly correlated with the infiltration of most immune cells (including B cells, CD8+ T cells, CD4+ T cells, neutrophils, monocytes, macrophages, and Treg cells). We also found the close correlation between the mRNA expressions of CBX family and immune cell surface markers in DLBCL. The poliovirus receptor (PVR)-like protein co-signaling network involves multiple immune checkpoint receptors, such as CD226 (DNAX accessory molecule-1, DNAM-1), CD96 (T cell activation, increased late expression (TACLILE)), CD112R (PVRIG), and TIGIT (T-cell immunoglobulin). They interact with their ligands CD155 (PVR/Necl-5), CD112 (PVRL2/nectin-2), CD111 (PVRL1/nectin-1), and CD113 (PVRL3/nectin-3), thereby regulating the function of immune cells (especially NK and T cells). These immune checkpoints have become potential targets for tumor immunotherapy in recent years [47–50]. Moreover, PD-1/PD-L1 immunotherapy had significant clinical progress in various cancers (including DLBCL). Immunotherapy targeted the TIGIT-CD96-CD112R-CD226 axis and PD-1/PD-L1 blocking therapy could play a synergistic role in tumor treatment [48, 51, 52]. Surprisingly, our research found that the mRNA expressions of CBX family members in DLBCL were significantly correlated with the expression levels of most immune cell surface markers, especially the widely studied PVR-like protein receptors/ ligands and PDL-1 immune checkpoint. For example, it was clear that CBX1/5/6 were significantly positively correlated with PDL-1 in Table 2 (correlation coefficient > 0.5).

Drug sensitivity analysis provided a number of small molecule compounds that were sensitive to DLBCL cells with high CBX family expression. We found that DLBCL cells with high CBX1 expression appeared to be resistant to common antitumor drugs (Fig. 8). However, there was a polarized difference in the drug resistance of DLBCL cells with high CBX2/5 expression. DLBCL cells were resistant to some of common antitumor drugs such as Trametinib, Selumetinib, Refametinib, which were a class of molecules targeting ERK MAPK signaling (Table S1), but sensitive to anothers (such as Phenformin, Methotrexate, 5-Fluorouracil). This suggested to us that ERK MAPK signaling may be responsible for the drug sensitivity of DLBCL cells with high CBX2/5 expression, but further experimental studies are needed. The CBX family, for example, CBX4 has been shown to affect the resistance of hepatocellular carcinoma cells to sorafenib [53], the effect of other members on resistance needs to be further investigated, and these may be research directions to explain the development of drug resistance in DLBCL patients.

## Conclusion

In summary, we provided a detailed analysis of the relationship between the CBX family and the prognosis of DLBCL. Distinguished from other studies, We found that high mRNA expressions of CBX2/3/5/6 were associated with poor prognosis in DLBCL patients, and Multivariate COX regression indicated that CBX3 was independent prognostic marker. We performed immunohistochemistry to verify the expressions of CBX family in DLBCL tissues. Besides, our study also found an association between the CBX family and antitumour drug resistance, and provided a relationship between CBX family expression and immune cell infiltration.

## Supplementary Information

**Additional file 1:** **Figure S1. **The expression of CBX1/2/3/5/6/8 in Diffuse large B cell lymphoma (DLBCL) cell lines. (A) The expression of CBX family in human cancer cell lines (including DLBCL), analyzed by the CCLE dataset. (B) The expression of CBX family in DLBCL cell line, analyzed by the EMBL-EBI database.

**Additional file 2: Figure S2.** Forest plot of the multivariate Cox regression analysis of CBX1/2/5/6/8 in Diffuse Large B-cell Lymphoma (DLBCL). The threshold P-value was defined as 0.05.

**Additional file 3:** **Table S1. **Details of common existed small molecules or drugs.

## Data Availability

The databases used in this study are licensed, and the data supporting the results of this study are available from the corresponding author.

## Abbreviations

DLBCL: Diffuse Large B-cell Lymphoma
TME: Tumor microenvironment
CBX: Chromobox
RBPs: RNA-binding proteins
OS: Overall survival
GEPIA: Gene Expression Profiling Interactive Analysis
GO: The Gene Ontology
KEGG: The Kyoto Encyclopedia Gene and Genome
BP: Biological process
CC: Cellular component
MF: Molecular function
HPA: Human Protein Atlas
GEO: Gene Expression Omnibus
TIMER2.0: Tumor Immune Estimation Resource 2.0
AOD: Average optical density
Tregs: Regulatory T cells
TAMs: Tumor-associated macrophages
HCC: Hepatocellular Carcinoma
DFS: Disease-free survival; HR, hazard ratio
