# Supplementary material for: An integrative bioinformatics investigation and experimental validation of chromobox family in diffuse large B-cell lymphoma
Source: BMC Cancer. 2023 Jul 10;23:641. doi: 10.1186/s12885-023-11108-6 (PMC10331996; doi:10.1186/s12885-023-11108-6)
Supplement: Supplementary file 1 — Additional file 1: Figure S1. The expression of CBX1/2/3/5/6/8 in Diffuse large B cell lymphoma (DLBCL) cell lines. (A) The expression of CBX family in human cancer cell lines (including DLBCL), analyzed by the CCLE dataset. (B) The expression of CBX family in DLBCL cell line, analyzed by the EMBL-EBI database. [file 12885_2023_11108_MOESM1_ESM.pdf]

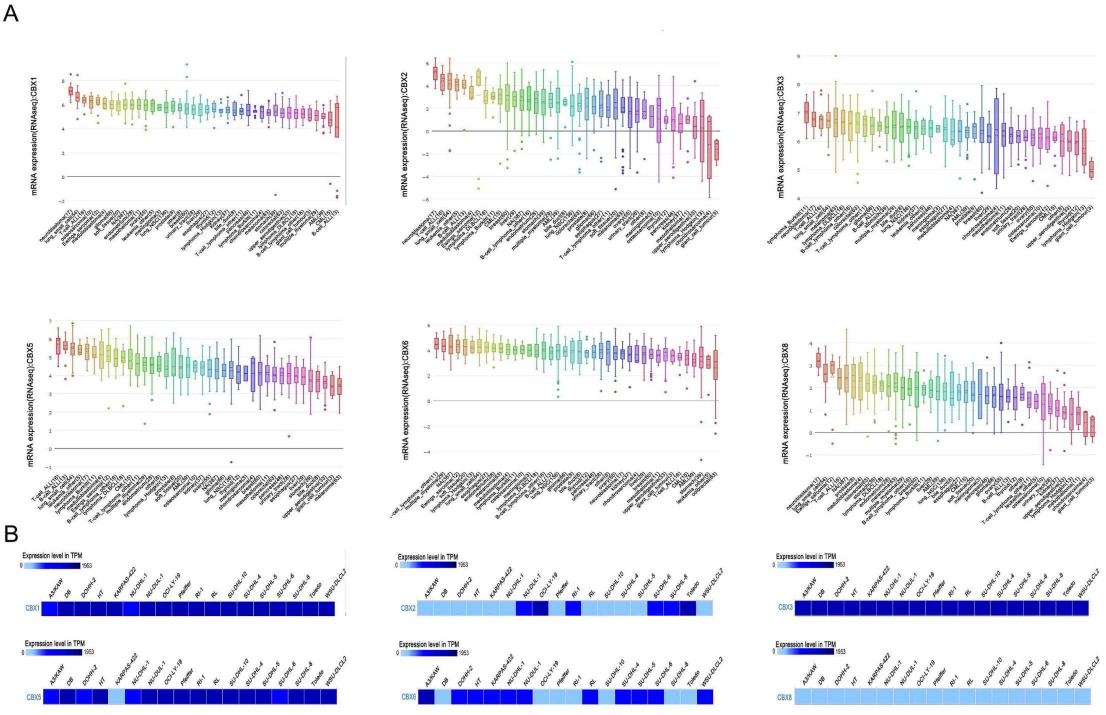

Figure S1. The expression of CBX1/2/3/5/6/8 in Diffuse large B cell lymphoma (DLBCL) cell lines. (A) The expression of CBX family in human cancer cell lines (including DLBCL), analyzed by the CCLE dataset. (B) The expression of CBX family in DLBCL cell line, analyzed by the EMBL-EBI database.
