# Supplementary material for: An integrative bioinformatics investigation and experimental validation of chromobox family in diffuse large B-cell lymphoma
Source: BMC Cancer. 2023 Jul 10;23:641. doi: 10.1186/s12885-023-11108-6 (PMC10331996; doi:10.1186/s12885-023-11108-6)
Supplement: Supplementary file 2 — Additional file 2: Figure S2. Forest plot of the multivariate Cox regression analysis of CBX1/2/5/6/8 in Diffuse Large B-cell Lymphoma (DLBCL). The threshold P-value was defined as 0.05. [file 12885_2023_11108_MOESM2_ESM.pdf]

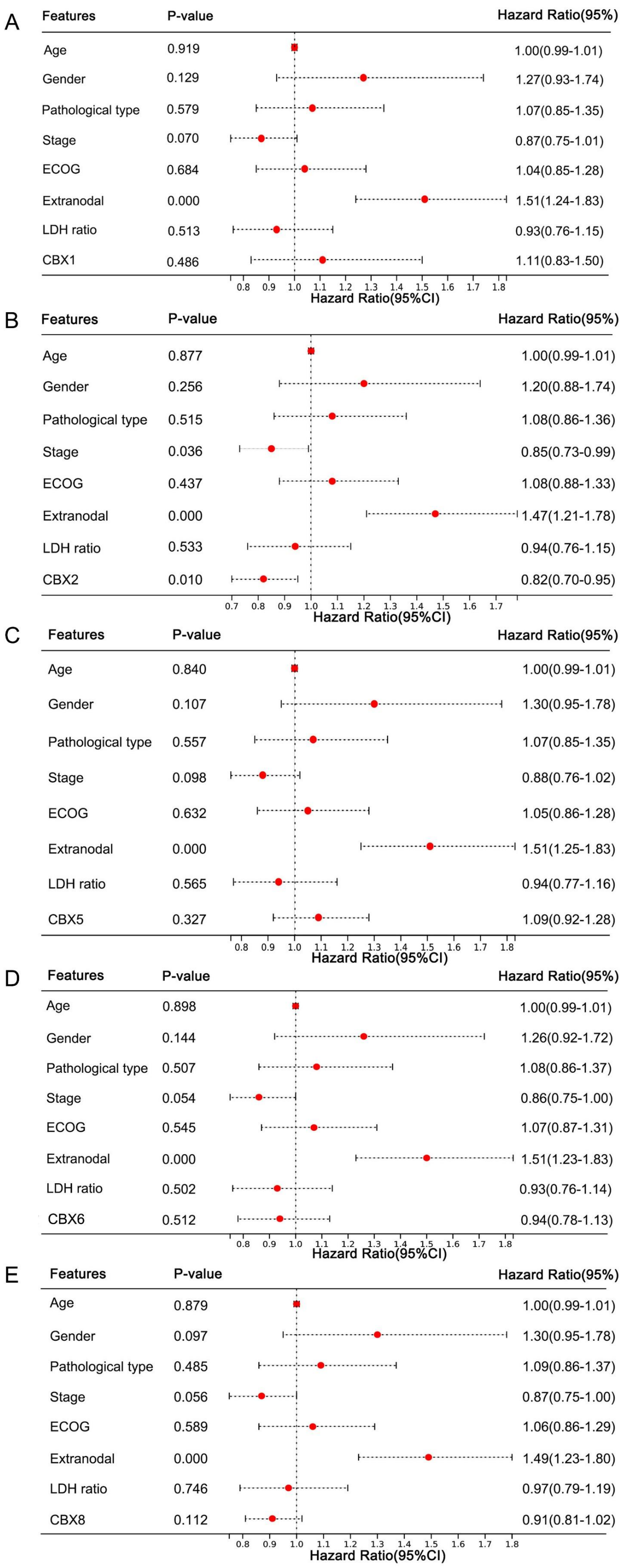

Figure S2. Forest plot of the multivariate Cox regression analysis of CBX1/2/5/6/8 in Diffuse Large B-cell Lymphoma (DLBCL). The threshold P-value was defined as 0.05.
