# Supplementary material for: An integrative bioinformatics investigation and experimental validation of chromobox family in diffuse large B-cell lymphoma
Source: BMC Cancer. 2023 Jul 10;23:641. doi: 10.1186/s12885-023-11108-6 (PMC10331996; doi:10.1186/s12885-023-11108-6)
Supplement: Supplementary file 3 — Additional file 3: Table S1. Details of common existed small molecules or drugs. [file 12885_2023_11108_MOESM3_ESM.docx]

**Table S1.** Details of common existed small molecules or drugs.

| Drug_name | Synonyms | Drug targets | Drug targets pathway |
| --- | --- | --- | --- |
| Tanespimycin | 17-AAG, BMS-722782 | HSP90 | Protein stability and degradation |
| Trametinib | GSK1120212, Mekinist | MEK1, MEK2 | ERK MAPK signaling |
| (5Z)-7-Oxozeaenol | 5Z-7-Oxozeaenol, LL-Z1640-2 | TAK1 | Other, kinases |
| CI-1040 | CI 1040, PD-18435, PD-184352, 212631-79-3 | MEK1, MEK2 | ERK MAPK signaling |
| PD0325901 | PD-0325901, PD 0325901 | MEK1, MEK2 | ERK MAPK signaling |
| Refametinib | RDEA119, BAY-86-9766, BAY 869766 | MEK1, MEK2 | ERK MAPK signaling |
| Z-LLNle-CHO | Z-L-Norleucine-CHO, Gamma-Secretase Inhibitor 1 | gamma-secretase | Other |
| Selumetinib | AZD6244, AZD-6244, ARRY-886 | MEK1, MEK2 | ERK MAPK signaling |
| AS601245 | - | JNK1, JNK2, JNK2 | JNK and p38 signaling |
| AZ628 | AZ-628, AZ 628 | BRAF | ERK MAPK signaling |
| CGP-60474 | KIN001-019, CGP60474, CGP 60474 | CDK1,CDK2,CDK5,CDK7,CDK9, PKC | Cell cycle |
| Dabrafenib | GSK2118436, Tafinlar | BRAF | ERK MAPK signaling |
| PLX-4720 | PLX4720, PLX 4720 | BRAF | ERK MAPK signaling |
| Rucaparib | PF-01367338, AG-014699, AG-14447, AG-14699 | PARP1, PARP2 | Genome integrity |
| JNK-9L | KIN001-204, JNK inhibitor 9l | JNK2, JNK3 | JNK and p38 signaling |
| JW-7-52-1 | NA | MTOR | PI3K/MTOR signaling |
| SB590885 | SB-590885 | BRAF | ERK MAPK signaling |
| Sunitinib | Sutent, Sunitinib Malate, SU-11248 | PDGFR, KIT, VEGFR, FLT3, RET, CSF1R | RTK signaling |
| TW 37 | TW37, TW-37 | BCL2, BCL-XL, MCL1 | Apoptosis regulation |
| Tipifarnib | Zarnestra | Farnesyl-transferase (FNTA) | Other |
| VX-11e | VX11e, VX11e | ERK2 | ERK MAPK signaling |
| XMD8-85 | ERK5-IN-1 | ERK5, BET | Other |
| AS605240 | KIN001-173, AS-605240 | PI3Kgamma | PI3K/MTOR signaling |
| Idelalisib | CAL-101, Zydelig | PI3Kdelta | PI3K/MTOR signaling |
| OSI-930 | OSI 930 OSI930 | KIT | RTK signaling |
| Pelitinib | EKB-569, EKB 569 | EGFR | EGFR signaling |
| AICA Ribonucleotide | AICAR, N1-(b-D-Ribofuranosyl)-5-aminoimidazole-4-carboxamide | AMPK agonist | Metabolism |
| AZD8055 | AZD-8055 | MTORC1, MTORC2 | PI3K/MTOR signaling |
| THZ-2-49 | - | CDK9 | Cell cycle |
| 5-Fluorouracil | 5-FU | Antimetabolite (DNA & RNA) | Other |
| NVP-BHG712 | BHG712 | EPHB4 | RTK signaling |
| Methotrexate | Abitrexate, Amethopterin, Rheumatrex, Trexall, Folex | Antimetabolite | DNA replication |
| Phenformin | DBI | Biguanide agent | Other |
| SB52334 | SB-52334, SB 52334 | ALK5 | Other, kinases |
